# New Records of *Ditylenchus* Species from Southern Alberta, Canada

**DOI:** 10.3390/plants12050998

**Published:** 2023-02-22

**Authors:** Maria Munawar, Atta Ur Rahman, Pablo Castillo, Dmytro P. Yevtushenko

**Affiliations:** 1 Department of Biological Sciences, University of Lethbridge, 4401 University Drive W, Lethbridge, AB T1K 3M4, Canada; 2 Institute for Sustainable Agriculture (IAS), Spanish National Research Council (CSIC), Campus de Excelencia Internacional Agrolimentario, ceiA3, Avda. Menendez Pidal s/n, 14004 Cordoba, Spain

**Keywords:** distribution, diversity, ecology, fungivores, identification, microscopy, molecular, stylet bearing, taxonomy, sequencing

## Abstract

The presence of plant-parasitic nematodes (PPNs) in cultivated areas is a limiting factor in achieving marketable crop yield. To control and alleviate the effects of these nematodes and determine appropriate management strategies, species-level identification is crucial. Therefore, we conducted a nematode diversity survey, which resulted in the detection of four *Ditylenchus* species in cultivated areas of southern Alberta, Canada. The recovered species had six lines in the lateral field, delicate stylets (>10 µm long), distinct postvulval uterine sacs, and pointed to rounded tail tips. The morphological and molecular characterization of these nematodes revealed their identity as *D. anchilisposomus*, *D. clarus*, *D. tenuidens* and *D. valveus*, all of which are members of the *D*. *triformis* group. All of the identified species were found to be new records in Canada except for *D. valveus*. Accurate *Ditylenchus* species identification is crucial because false-positive identification can result in the implementation of quarantine measures over the detected area. Our current study not only documented the presence of *Ditylenchus* species from southern Alberta, but also described their morpho-molecular characteristics and subsequent phylogenetic relationships with related species. The results of our study will aid in the decision on whether these species should become a part of nematode management programs since nontarget species can become pests due to changes in cropping patterns or climate.

## 1. Introduction

*Ditylenchus* Filipjev [1] is the largest genus of the family Anguinidae Nicoll, [2,3] that has adapted to a wide range of ecological processes, including phytoparasitism [4,5,6], mycophagy [7,8,9], phoretic association with insects in soil [10,11], biocontrol of weeds [12], and acting as a vector for *Corynebacterium* spp. [13,14,15]. The majority of studied *Ditylenchus* members are fungal feeders [3,16]. However, *D. africanus* Wendt, Swart, Vrain and Webster [17]; *D. angustus* (Butler) Filipjev [1,18]; *D. destructor* Thorne [19]; *D. dipsaci* (Kühn) Filipjev [1,20]; *D. gallaeformans* Oliveira, Santin, Seni, Dietrich, Salazar, Subbotin, Mundo-Ocampo, Goldenberg and Barreto [12]; *D. gigas* Vovlas, Troccoli, Palomares-Rius, De Luca, Liebanas, Landa, Subbotin and Castillo [6]; and *D. myceliophagus* Goodey [7] have attracted attention due to their parasitic potential and quarantine regulations [4,6,21,22,23,24]. Several *Ditylenchus* species are polyphagous and display endoparasitic behaviour; as a result, these species can be disseminated through seeds, plant material or contaminated field equipment [8,25,26].

The regulated status of *Ditylenchus* spurred widespread research, which led to the discovery of numerous new species without giving adequate consideration to the limits of species variability [27]. Several taxonomists have reviewed the genus characteristics in attempts to limit the number of valid species by revisions and synonymization [3,27,28]. More recently, Hashemi and Karegar [16] updated the entire genus *Ditylenchus* and documented 60 nominal species; presently, the genus contains 63 species. Of all valid species, the literature lists *D. filimus* Anderson [29] and *D. dryadis* Anderson and Mulvey [30] as Canadian native species, whereas *D. valveus* Thorne and Malek [31] and *D. weischeri* Chizhov, Borisov and Subbotin [32] were listed as new records from Canada. In addition, *D. destructor* Thorne [19] and *D. dipsaci* (Kühn, 1857) Filipjev [1,20] were reported from isolated locations (British Columbia, Ontario, and Prince Edward Island) and subjected to strict quarantine regulations to prevent spread [13,21,26,29,30,33,34,35,36]. Among the isolated species described in Canada, *D. dipsaci* is the only one reported from Alberta where it was found in alfalfa-growing areas [13]. Therefore, the cultivated areas of southern Alberta are regularly surveyed and examined for the presence of nematode pest species [37,38,39]. These surveys include detecting not only the regulated pest species but also new and unusual nematode species that may pose a challenge to agricultural produce. During our nematode inventory survey, we detected four different populations of *Ditylenchus* in no-till fields (in which soil was undisturbed from harvest to planting). Due to its regulatory importance, the recovered unidentified populations were immediately processed for morphological and molecular characterization. Light microscopy indicated the presence of delicate stylets (>10 µm long), six lateral lines and round to pointy tail tips. The populations were identified as members of the *D. triformis* group, which includes species with six lateral lines, a rounded tail tip, and a mycophagous life cycle.

Further morphological comparisons revealed their identity as *D. anchilisposomus* (Tarjan) Fortuner [28,40], *D. clarus* Thorne and Malek [31], *D. tenuidens* Gritsenko [41], and *D. valveus*. All of these species are new records in Canada except for the *D. valveus*.

The objectives of the present study were to: (1) document the presence of *Ditylenchus* species from cultivated areas in southern Alberta, (2) provide detailed morphological and molecular characterizations of these species, and (3) study the phylogenetic relationship of these species with related ones. The results of this study will aid in the decision of whether these species should be incorporated into nematode management programs since nontarget species can become pests due to changes in cropping patterns or climate.

## 2. Results

### 2.1. Description of Ditylenchus anchilisposomus (Tarjan) Fortuner [28,40] 

**Figure 1:**
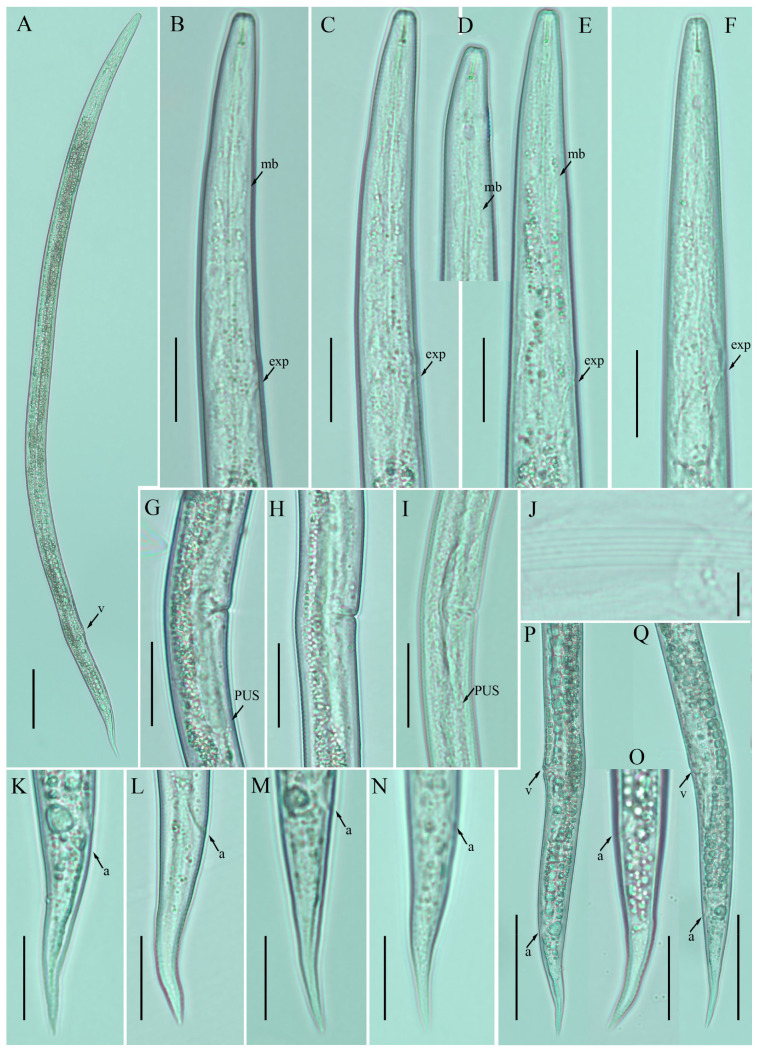
Photomicrographs of female *Ditylenchus anchilisposomus* (Tarjan) Fortuner [28,40] (**A**) Entire body; (**B**–**F**) pharyngeal regions; (**G**–**I**) vulval regions; (**J**) lateral field lines; (**K**–**O**) tail regions; (**P**,**Q**) posterior body to tail terminus. Scale bars: (**A**,**P**,**Q**) 50 μm; (**B**–**I**,**K**–**O**) 20 μm; (**J**) 5 μm. Arrows: (a) anus; (exp) secretory–excretory pore; (mb) median bulb; (PUS) postvulval uterine sac; (v) vulva.

*Female:* Body cylindrical, slightly ventral arcuate when heat-relaxed. Cuticle finely annulated; lateral field with six incisures. Lip region low, anteriorly truncated, with rounded margins, continuous with body contour. Stylet delicate; conus 30–35% of total stylet length. Stylet knobs small and rounded. Dorsal pharyngeal gland orifice (DGO) situated close to stylet knobs. Median pharyngeal bulb moderately developed, with indistinct elongated valve plates. Isthmus slender, encircled with a nerve ring. Hemizonid streak-like, two to three annuli anterior to the secretory–excretory pore. This pore located slightly in the range of the anterior level of the pharyngeal basal bulb. Basal pharyngeal bulb pyriform to elongated, slightly overlapping intestine. Ovary outstretched, oocytes arranged in a single row, spermatheca tubular devoid of sperm, in line with the genital tract. Vagina straight; vulva a transverse slit occupying less than half of the corresponding body width. Vulval lips simple, not protruding. Post-vulval uterine sac empty; tube-like sac along the ventral body wall ca. more than one vulval body width long. Anus a transverse slit. Tail conical; the posterior half of the tail curved ventrally, giving rise to a rounded to pointed terminus (Figure 1; Table 1).

*Male*: not detected. 

*Remarks*: *Ditylenchus anchilisposomus* was originally described from California, USA in the rhizosphere of grass as *Pseudhalenchus anchilisposomus* [40]. This genus was formed to accommodate the species that were closely related to the genera, *Ditylenchus*, *Halenchus* Cobb [42] and *Tylenchus* Bastian [43]. Taxonomically, *Pseudhalenchus* Tarjan [40] can be differentiated from *Ditylenchus* by having overlapped esophageal glands. Thorne [44] studied *D. destructor* and reported that the position of the pharyngeal lobe is a host-dependent character. Observations of *D. myceliophagus* also suggest that the length of the pharyngeal overlap varies significantly during the life cycle of an individual [28]. Consequently, *P. anchilisposomus* was transferred to the genus, *Ditylenchus,* as *D. anchilisposomus* by Fortuner [28]. After the formal recognition, the species was reported from Nebraska, USA and Iran without indication of the host association [16,31]. In the present study, we found *D. anchilisposomus* in a no-till field (previously planted with potatoes) from southern Alberta. The description of the Nebraska population provided the measurements of a single specimen, and, therefore, cannot be used for intraspecies variation comparison. Moreover, the description from Iran is devoid of photo documentation or illustrations. Alternately, our *D. anchilisposomus* population provides a complete set of morphological and morphometric characteristics, which are consistent with the original description’s characteristics and can be used for further molecular and taxonomical considerations (Table 1). 

### 2.2. Description of Ditylenchus clarus Thorne and Malek [31] 

**Figure 2:**
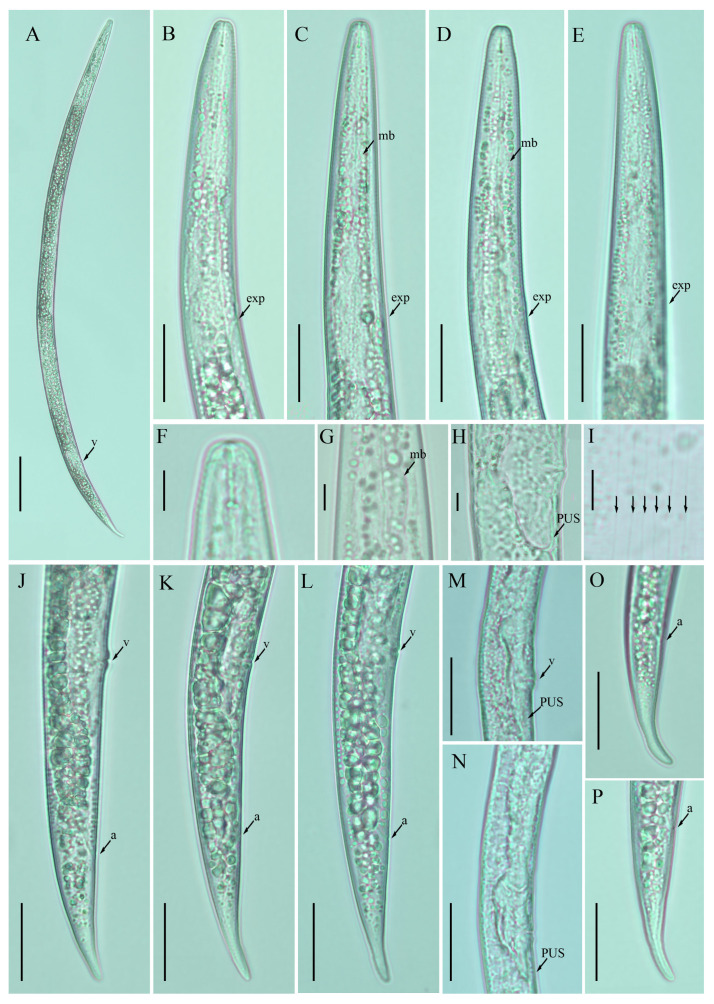
Photomicrographs of female *Ditylenchus clarus* Thorne and Malek [31] (**A**) Entire body; (**B**–**E**) pharyngeal regions; (**F**) lip region; (**G**) median bulb; (**H**,**M**,**N**) vulval regions; (**I**) lateral field lines; (**J**–**L**) posterior body to tail terminus; (**O**,**P**) tail regions. Scale bars: (**A**) 50 μm; (**B**–**E**,**J**–**P**) 20 μm; (**F**–**I**) 5 μm. Arrows: (a) anus; (exp) secretory–excretory pore; (mb) median bulb; (PUS) postvulval uterine sac; (v) vulva.

*Female:* Body cylindrical, medium-sized, slightly ventral arcuate when heat-relaxed. Cuticle finely annulated; lateral field with six incisures. Lip region low, anteriorly flattened, the width is twice the height of the lip region. Stylet delicate; conus 30–40% of total stylet length. Stylet knobs small and rounded. Dorsal pharyngeal gland orifice (DGO) situated close to stylet knobs. Median pharyngeal bulb moderately developed with elongated valve plates. Isthmus slender encircled with a nerve ring. Hemizonid streak-like, one to three annuli anterior to the secretory–excretory pore. This pore located in the range of the pharyngeal basal bulb. Basal pharyngeal bulb pyriform to elongated, slightly overlapping intestine. Ovary outstretched in mature females, extending forward to the median bulb. Oocytes in a single row, columella well discernible, spermatheca tubular devoid of sperm, in line with the genital tract. A valvular apparatus present at the entrance to the uterine tract. Vagina straight; vulva a transverse slit occupying less than half of the corresponding body width. Vulval lips prominent slightly protruding in some specimens. Postvulval uterine sac small, empty, broad sac along the ventral body wall, extending ca. halfway to anus in some specimens. Anus a transverse slit. Tail conoid, stout; the posterior half of the tail curved ventrally, giving rise to a rounded terminus (Figure 2). 

*Male:* not detected.

*Remarks:* The species was originally described from a thicket in South Dakota, USA, without definitive host association [31]. In the present study, *D. clarus* was isolated from *Amaranthus* sp. rhizosphere growing on a headland of a post harvested wheat field. The general appearance and dimensions of the southern Alberta population of *D. clarus* were consistent with the original description except for the shorter stylet length (7.0–7.5 vs. 10 µm). After the formal description, the species was not reported again. Moreover, the original description likely provided the measurements of a single specimen and, therefore, cannot be used for intraspecies variation comparison. In contrast, southern Alberta population data can be used for further molecular and taxonomical considerations (Table 2). 

### 2.3. Description of Ditylenchus tenuidens Gritsenko [41]

*Female*: Body cylindrical, moderately long, slightly ventral arcuate when heat-relaxed. Cuticle finely annulated, lateral field with six incisures. Lip region narrow anteriorly flattened; width is twice the height of the lip region. Stylet delicate; conus 35–40% of total stylet length. Stylet knobs small, rounded, anteriorly sloping. Dorsal pharyngeal gland orifice (DGO) situated close to stylet knobs. Median pharyngeal bulb moderately developed with elongated valve plates. Isthmus slender encircled with a nerve ring. Hemizonid streak-like, one to three annuli anterior to the secretory–excretory pore. This pore located slightly anterior to or in the range of the anterior level of the pharyngeal basal bulb. Basal pharyngeal bulb pyriform to slightly elongated, abutting intestine. Ovary outstretched, oocytes in a single row, spermatheca and columella well discernible. Spermatheca rounded to elongate; in a few specimens, scarcely filled with rounded sperm generally in line with the genital tract. A valvular structure surrounds the entrance to the uterine tract. Vagina straight; vulva a transverse slit occupying less than half of the corresponding body width. Vulval lips simple, not protruding. Postvulval uterine sac empty tube-like, along the ventral body wall, one or more vulval body width long. Anus a transverse slit. Tail cylindrical elongated; the posterior half of the tail slender and tapers to form a pointed terminus.

*Male*: Body slightly shorter and more cylindroid than females. The anterior region is similar to females. Bursa leptoderan, starting at the same level as the anterior end of the spicules and extends less than half of the tail length. Spicules arcuate moderately long; gubernaculum simple embedded in cloacal sac. Cloacal opening smooth; tail shape is similar to that of females.

**Figure 3:**
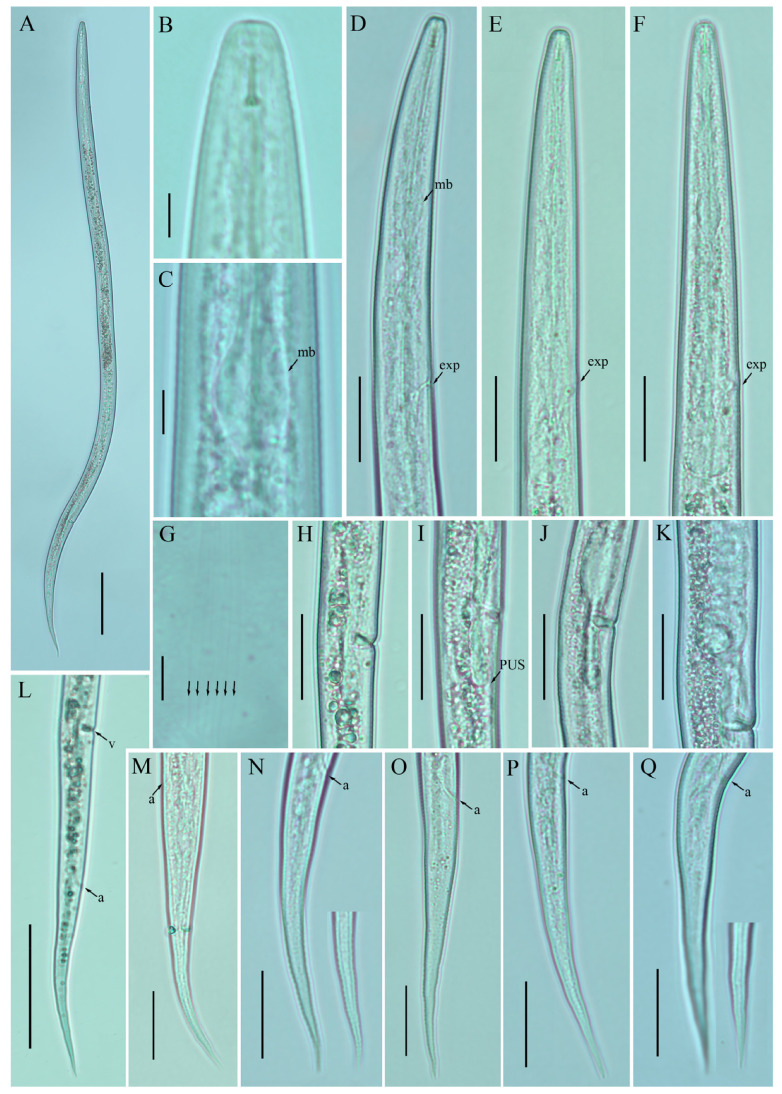
Photomicrographs of female *Ditylenchus tenuidens* Gritsenko [41] (**A**) Entire body; (**B**) lip region; (**C**) median bulb; (**D**–**F**) pharyngeal regions; (**G**) lateral field lines; (**H**–**K**) vulval regions; (**L**) posterior body to tail terminus; (**M**–**Q**) tail regions. Scale bars: (**A**,**L**) 50 μm; (**B**,**C**,**G**) 5 μm; (**D**–**F**,**H**–**K**,**M**–**Q**) 20 μm. Arrows: (a) anus; (exp) secretory–excretory pore; (mb) median bulb; (PUS) postvulval uterine sac; (v) vulva.

**Figure 4:**
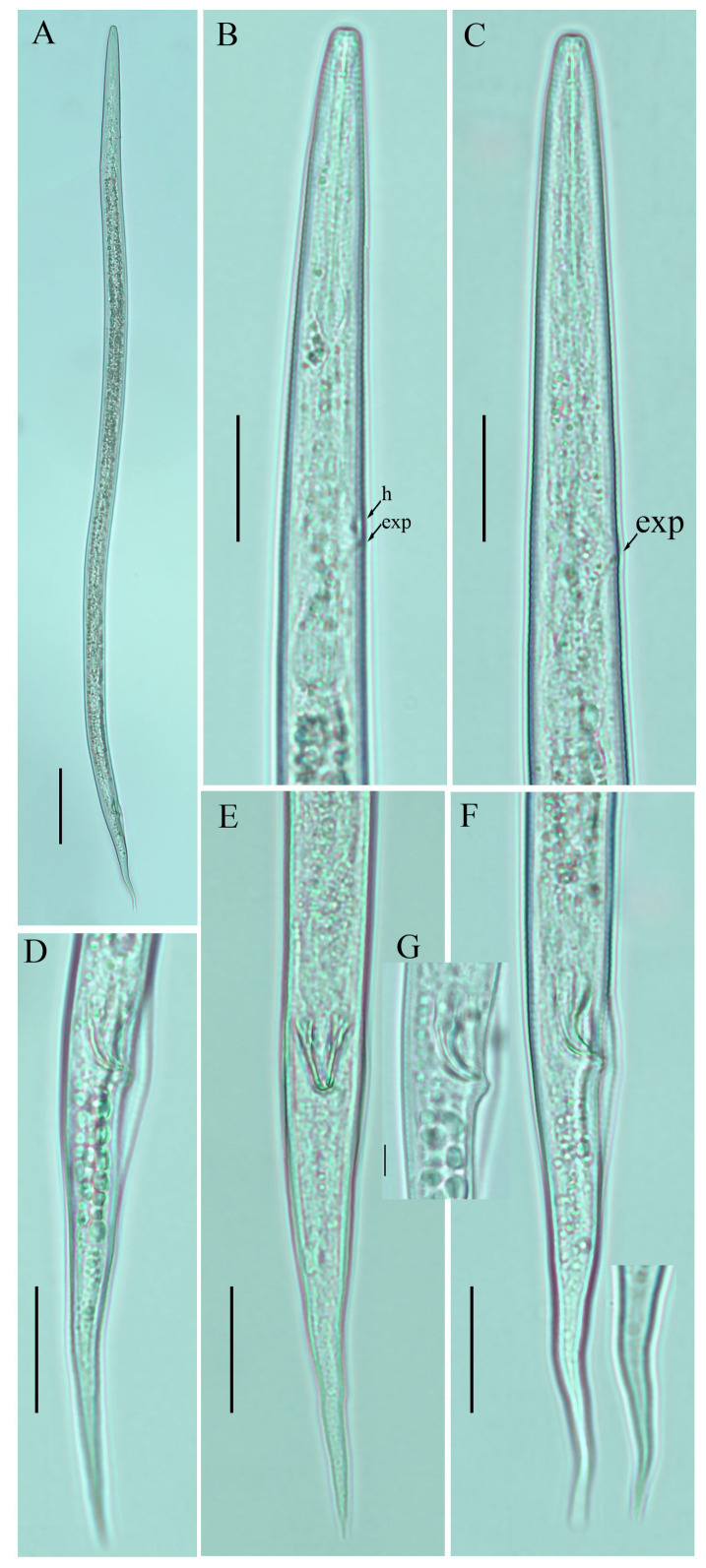
Photomicrographs of male *Ditylenchus tenuidens* Gritsenko [41] (**A**) Entire body; (**B**,**C**) pharyngeal region; (**D**–**F**) tail region; (**G**) spicule. Scale bars: (**A**) 50 μm; (**B**–**F**) 20 μm; (**G**) 5 μm. Arrows: (h) hemizonid; (exp) secretory–excretory pore.

*Remarks*: *Ditylenchus tenuidens* was originally described from Kyrgyzstan in the rhizospheres of potato and wheat [41]. After the formal description, the species was reported from Poland and Sweden in leaf litter, forest organic soils, and moss samples by Brzeski [27]. Two decades later, Mirshekari and Abdollahi [45] found the same species in wheat and vegetable fields in Iran; however, the authors did not mention if the samples were collected before planting or after harvest. In the present study, we found *D. tenuidens* in a no-till field (previously planted with grains). The morphological and morphometric characteristics of the southern Alberta *D. tenuidens* population matched well with the original and subsequent descriptions except for a slightly longer body length and tail in the former population. Studies on the food source of *D. destructor* and *D. myceliophagous* found that the body length depended on the food supply, in particular the fungal species on which they fed [46,47]. Moreover, the body lengths correlated with the tail lengths [28]. Considering this, the slightly longer bodies and tail lengths of the southern Alberta population can be considered intraspecies variation. Bionomically, *D. tenuidens* have been reported from different habitats, which suggests that the host preference of *D. tenuidens* is quite diverse (agronomic crops, leaf litter and moss). The description provided by Brzeski [27] is taxonomically adequate, however, lacks photo documentation; the Iranian description [45] is based on illustrations and is in the author’s native language (Farsi). Here, we described the southern Alberta *D. tenuidens* population using the integrative taxonomical approach, making these data suitable for further molecular and taxonomical considerations (Figure 3 and Figure 4; Table 3 and Table 4).

### 2.4. Description of Ditylenchus valveus Thorne and Malek [31]

*Female:* Body cylindrical, moderately long, slightly ventral arcuate when heat-relaxed. Cuticle finely annulated, lateral field with six incisures. Lip region low anteriorly flattened; width is twice the height of the lip region. Stylet delicate; conus 40–45% of total stylet length. Stylet knobs small, rounded, anteriorly sloping. Dorsal pharyngeal gland orifice (DGO) situated close to stylet knobs. Median pharyngeal bulb moderately developed with elongated valve plates. Isthmus slender encircled with a nerve ring. Hemizonid streak-like, one to three annuli anterior to the secretory–excretory pore. This pore located slightly anterior to or in the range of the anterior level of the pharyngeal basal bulb. Basal pharyngeal bulb pyriform to slightly elongated, abutting intestine. Ovary outstretched, reflexed in old females, extending forward to the median bulb. Oocytes in a single row, spermatheca and columella well discernible. Spermatheca, scarcely filled with rounded sperm, generally in line with the genital tract

A conspicuous valvular apparatus surrounds the entrance to the uterine tract. Vagina straight; t vulva a transverse slit occupying less than half of the corresponding body width. Vulval lips prominent and slightly protruding in some specimens. Post-vulval uterine sac appears as an empty broad sac along the ventral body wall ca. one vulval body width long. Anus a transverse slit. Tail cylindrical and elongated; the posterior half of the tail curved ventrally, giving rise to variable tail tips, i.e., rounded, slightly pointed, and pointed.

*Male*: Body slightly shorter and more cylindroid than females. The anterior region is similar to females. Bursa leptoderan, starting at the same level as the anterior end of the spicules, and extends about half of the tail length. Spicules arcuate, moderately long; gubernaculum simple embedded in cloacal sac. Cloacal opening smooth; tail shape is similar to that of females.

**Figure 5:**
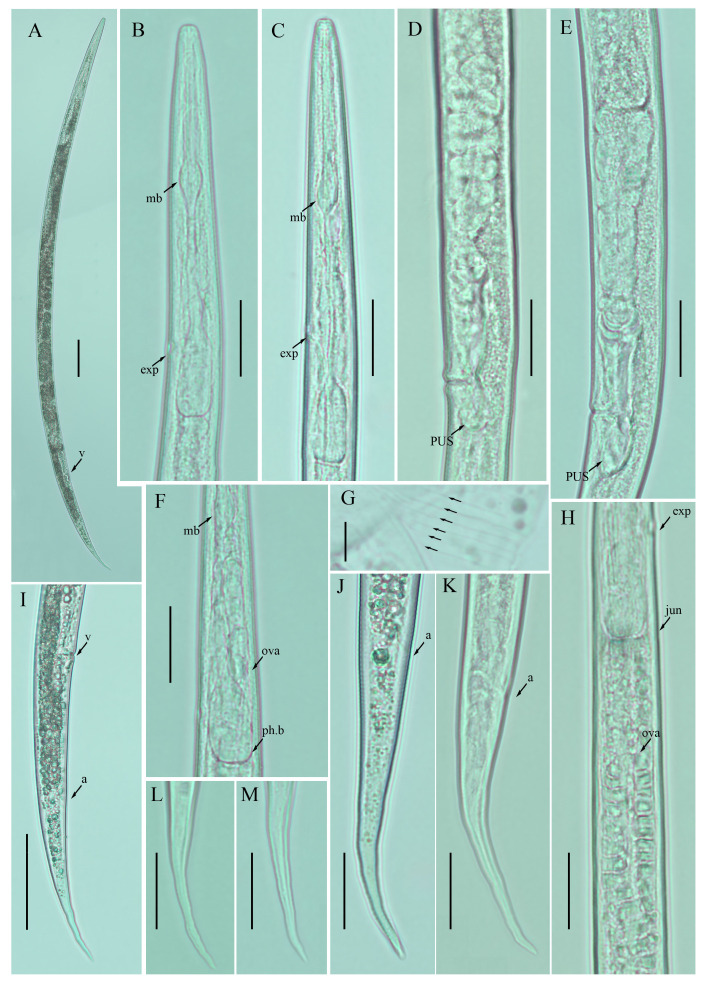
Photomicrographs of female *Ditylenchus valveus* Thorne and Malek [31] (**A**) Entire body; (**B**,**C**) pharyngeal region; (**D**,**E**) vulval regions; (**F**) posterior pharyngeal region; (**G**) lateral field lines; (**H**) post pharyngeal region showing reflexed ovary; (**I**) posterior body to tail terminus; (**J**,**K**) tail regions; (**L**,**M**) tail tips. Scale bars: (**A**,**I**) 50 μm; (**B**–**F**,**H**,**J**–**M**) 20 μm; (**G**) 5 μm. Arrows: (a) anus; (exp) secretory–excretory pore; (ph.b) pharyngeal bulb; (jun) pharyngeal intestinal junction; (mb) median bulb; (ova) ovary; (PUS) postvulval uterine sac; (v) vulva.

**Figure 6:**
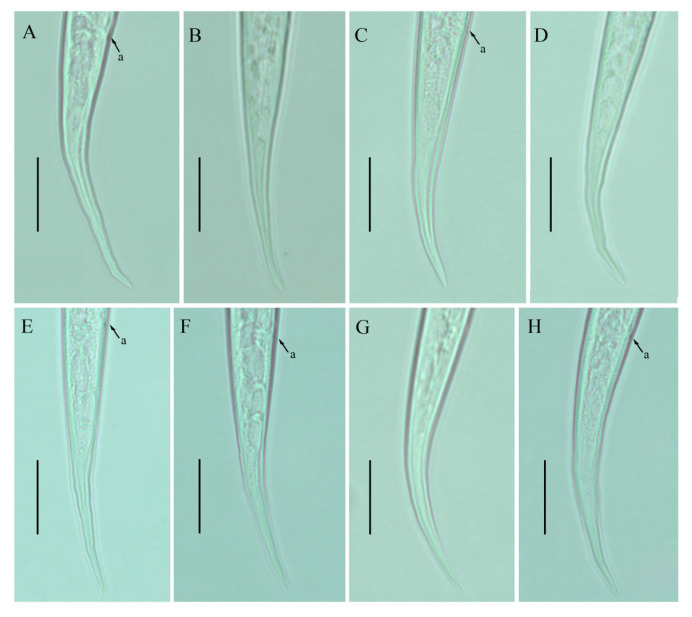
Photomicrographs of female *Ditylenchus valveus* Thorne and Malek [31] tail region variations (**A**–**H**) tail terminus rounded to pointed. Scale bars: 20 μm. Arrows: (a) anus.

**Figure 7:**
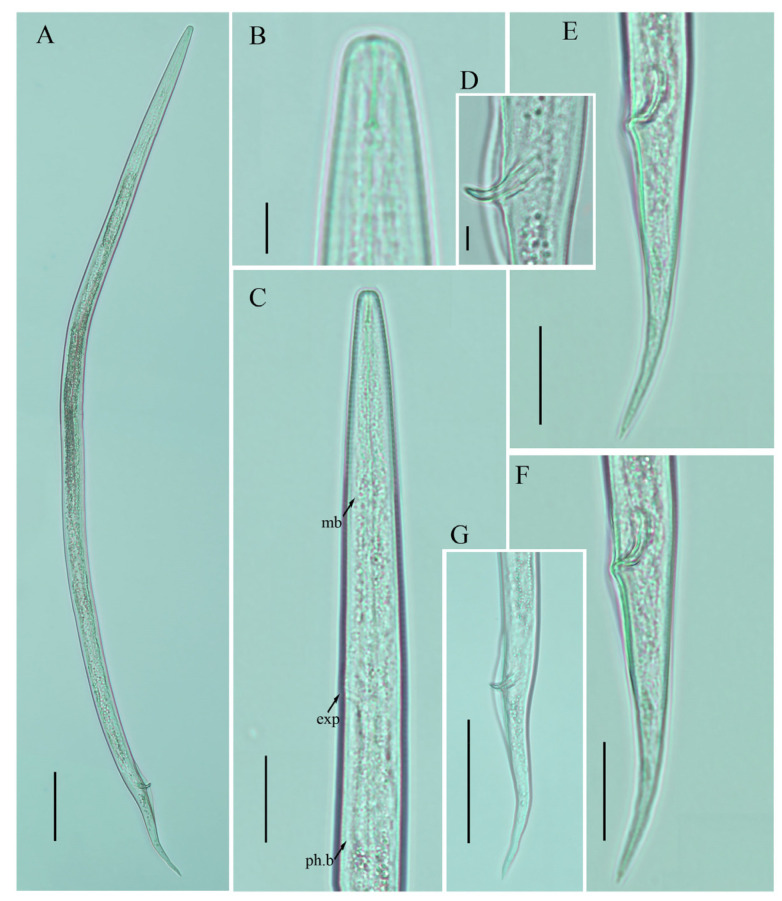
Photomicrographs of male *Ditylenchus valveus* Thorne and Malek [31] (**A**) Entire body; (**B**) lip region; (**C**) pharyngeal region; (**D**) spicule; (**E**–**G**) tail regions. Scale bars: (**A**,**G**) 50 μm; (**C**,**E**,**F**) 20 μm; (**B**,**D**) 5 μm. Arrows: (exp) secretory–excretory pore; (mb) median bulb; (ph.b) pharyngeal bulb.

*Remarks*: *Ditylenchus valveus* was originally reported from a ploughed field in South Dakota, USA [31]. After the formal description, the species was reported from the rhizospheres of unknown bushes (Poland, Bulgaria [27]) and olive trees (Iran [16,48]). In Canada, the species was previously reported from Manitoba in mushroom compost [29], whereas in this study we found *D. valveus* in a no-till field (previously planted with barley). The original description mentioned the presence of a conspicuous valvular structure at the entrance to the uterus, longer and reflexed ovaries, reaching past the basal pharyngeal bulb. These reproductive system characteristics were not observed in any of the subsequent descriptions except for our southernAlbertan population (see the female description section Figure 5, Figure 6 and Figure 7; Table 5 and Table 6). Morphometrically, the Bulgarian population of *D. valveus* is the smallest population as compared with the original and all other descriptions [27]. The population from Iran [48] has an unusually long postvulval uterine sac (PUS). Consequently, the morphology and morphometric characteristics of the *D. valveus* population from southern Alberta fit more precisely with the characteristics presented in the original description. 

### 2.5. Molecular Characterisation and Phylogeny of the Recovered Ditylenchus Species

We molecularly characterized the *Ditylenchus* species recovered in our survey using the 18S, D2–D3 of 28S and ITS rDNA diagnostic genes. We deposited the obtained sequences into the NCBI database under the following accession numbers: *D. anchilisposomus* (partial 18S, OP854643–OP854644; D2–D3 of 28S, OP854653–OP854654; and ITS, OQ058851–OQ058852); *D. clarus* (partial 18S, OP854645–OP854646; D2–D3 of 28S, OP854655–OP854656; and ITS, OQ058853–OQ058854); *D. tenuidens* (D2–D3 of 28S, OP854657–OP854658; and ITS, OQ058855–OQ058856); and *D. valveus* (partial 18S, OP854647–OP854652; D2–D3 of 28S, OP854659–OP854665; and ITS, OQ058857–OQ058861).

**Figure 8:**
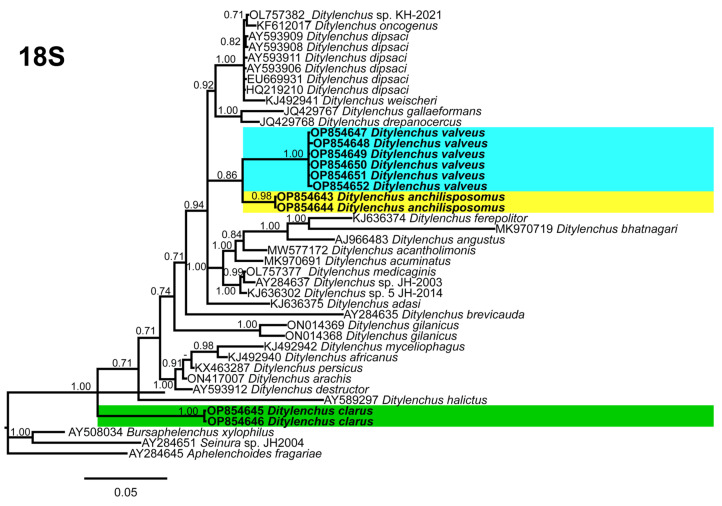
Phylogenetic relationships of the Canadian population of *Ditylenchus* species with related species. Bayesian 50% majority rule consensus tree as inferred from 18S rRNA sequence alignment under the general time−reversible model with invariable sites and a gamma−shaped distribution (GTR + G + I). Posterior probabilities of greater than 0.70 are provided for the corresponding appropriate clades. The sequences produced in this study are shown in bold, and the colored boxes indicate the clade association of the recovered *Ditylenchus* species.

**Figure 9:**
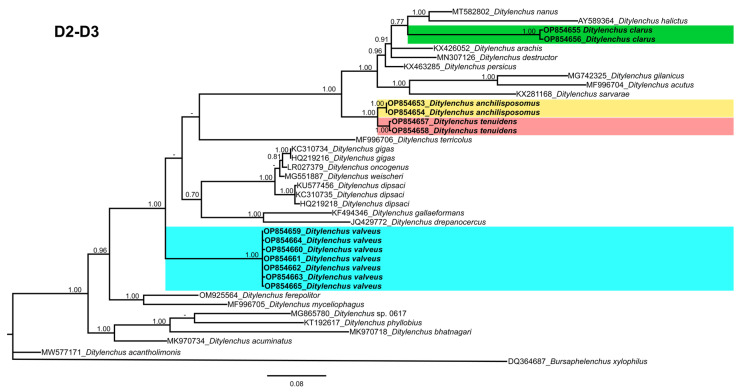
Phylogenetic relationships of the Canadian population of *Ditylenchus* species with related species. Bayesian 50% majority rule consensus tree as inferred from 28S rRNA sequence alignment under the general time−reversible model with invariable sites and a gamma−shaped distribution (GTR + G + I). Posterior probabilities of greater than 0.70 are provided for the corresponding clades. The sequences produced in this study are shown in bold, and the colored boxes indicate the clade association of the recovered *Ditylenchus* species.

**Figure 10:**
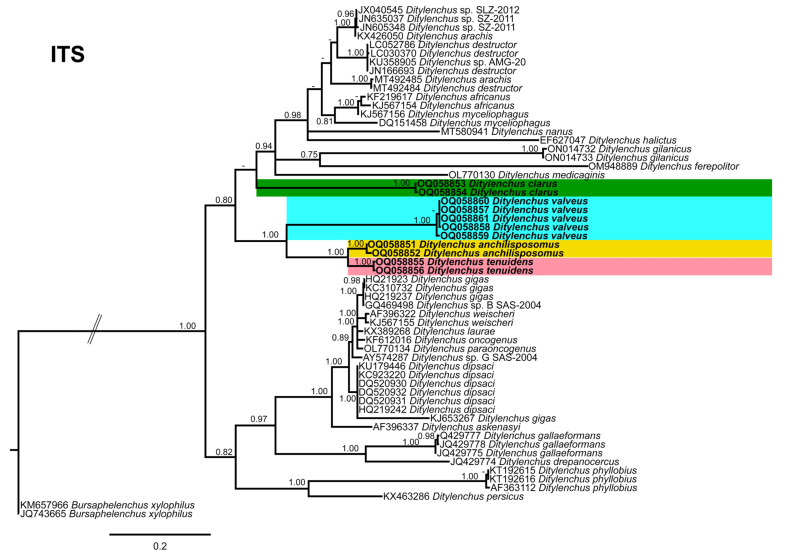
Phylogenetic relationships of the Canadian population of *Ditylenchus* species with related species. Bayesian 50% majority rule consensus tree as inferred from ITS rRNA sequence alignment under a transition model with a gamma−shaped distribution (TIM2 + G). Posterior probabilities of greater than 0.70 are provided for the corresponding clades. The sequences produced in this study are shown in bold, and the colored boxes indicate the clade association of recovered the *Ditylenchus* species.

Phylogenetic trees based on the 18S, D2–D3 of 28S and ITS rDNA genes were constructed to study the phylogenetic relationships of the recovered species with the available *Ditylenchus* species. The 18S tree (alignment 1694 bp; Figure 8) was constructed with 39 *Ditylenchus* sequences and *Bursaphelenchus xylophilus* Steiner and Buhrer (Nickle [49,50]) (AY508034), *Seinura* sp. (AY284251) and *Aphelenchoides fragariae* (Ritzema Bos) Christie, [51,52] (AY284545) as outgroup taxa. In this tree, *D. anchilisposomus* and *D. valveus* form a separate, moderately supporting clade (PP = 0.86) among other *Ditylenchus* species, and *D. clarus* occupies a basal position as a separate, well-supported clade (PP = 1.00). The 28S tree (alignment 769 bp; Figure 9) was constructed with 38 *Ditylenchus* sequences with *B. xylophilus* (DQ364687) as an outgroup taxon. In this tree, *D. clarus* shares a branch with *D. halictus* Giblin-Davis, Erteld, Kanzaki, Ye, Zeng and Center [10], (AY589364) and *D. nanus* Siddiqi [53](MT582802) in a low support (PP = 77%), whereas *D. anchilisposomus*, *D. tenuidens,* and *D. valveus* grouped independently among other *Ditylenchus* species in well-supported clades (PP = 1.00). The ITS tree (alignment 786 bp: Figure 10) was constructed from 57 *Ditylenchus* sequences and *B. xylophilus* (KM657966, JQ743665) as an outgroup taxon. In the ITS tree, *D. anchilisposomus*, *D. tenuidens* and *D. clarus* clustered together in a well-supported clade (PP = 1.00), whereas *D. valveus* clustered in a separate low-supported clade (PP = 0.68), maintaining a middle position in the tree; however, all these species grouped independently among other *Ditylenchus* species. 

Since the recovered species are distinctly different from each other, and do not share striking phylogenetic affinity with other known species, we excluded the sequence similarity comparison for this dataset. The species recovered in this study all belong to the *D*. *triformis* group; however, this grouping is not evident in the phylogenetic analysis, which supports the notion that morphological grouping aids in prompt identification but does not reflect distinct lineages [54]. Species in the genus *Ditylenchus* have been reported from diverse hosts and different geographic locations; for most species, the biology and their association with the hosts are unknown. We speculate that the species bionomics has some role in the phylogenetic status, and the availability of genus-wide sequencing data will unequivocally elucidate the relationships of these species with each other. 

## 3. Discussion

Based on morphological characteristics and bionomics, Siddiqi [3] divided *Ditylenchus* species into two main groups, namely, a *D*. *dipsaci* group (having four lateral lines, sharply pointed tails, and obligate parasitic behaviour) and a *D*. *triformis* group (having six lateral lines, rounded tail tips, and mainly fungal feeders). The *Ditylenchus* species recovered in this study belong to the *D*. *triformis* group; however, at present, we are unable to ascertain any details relating to these species’ host association, dispersal, and survival. We found these species in no-till fields; therefore, it is unknown if these nematodes were feeding on soil fungal propagules or surviving on crop residues or weeds. The native geographic range for *D. anchilisposomus*, *D. clarus*, and *D. valveus* of Western USA [31,40] and Kyrgyzstan for *D. tenuidens* [41] is now increased with the present records. The long-distance dispersal of these species is only possible through either phoretic association with insect vectors or transportation by humans and animals. There are reports of bark beetles and sweat bees acting as vectors for *Ditylenchus* species [10,11] and a presumption that many soil-inhabiting nematodes were introduced to different locations by migratory bison and birds in mud adhered to the animals [44,55]. For the moment, we do not have evidence to support the dispersal theories for these *Ditylenchus* species; we believe it is an area that warrants future research. Since the recovered species were found in moderate numbers (29–47 individuals in 250 g of soil), we do not consider them as pest species. However, it is crucial to monitor the quantities of *D. anchilisposomus*, *D. clarus*, *D. tenuidens,* and *D. valveus* under different crop rotation regimes, as Hajihassani et al. [26] demonstrated that the severity of disease development in peas and beans is closely related to the number of *D. dipsaci* infesting the plant.

Root lesion nematodes are dominating species in southern Alberta and have been reported from several cultivated areas [56]. In the present study, we found *Ditylenchus* species coexisting with root lesion, pin, stunt, and several other soil-inhabiting nematodes (members of Tylenchidae Orley [57], rhabditids, and dorylaimids). Root lesion nematodes are the third, and stem nematodes are the fifth most damaging PPNs worldwide [58]. Both these nematodes are migratory endoparasites; if left unchecked, they can affect plant growth and yields under elevated stress. In addition, the co-occurrence of several PPN groups can lay the foundation for a novel encounter between different nematode species and host plants, thus creating new disease complexes that may challenge host defenses [59]. *Ditylenchus* penetration and movement within a plant tissue have been shown to cause mechanical injuries that interfere with the uptake of water and nutrients [15]. Therefore, we emphasize the need for increased surveys and optimum agronomic practices to evaluate the PPN pre-planting numbers to prevent the subsequent spread of different PPN groups.

Recent phylogenetic studies do not concur on the paraphyletic status and presence of different lineages in *Ditylenchus* [10,11,60]. In our study, we noted that phylogenetic inference is effective for separating species, but with limited sequence-based information, these inferences are inconclusive in assigning species groups (*D*. *dipsaci* or *D*. *triformis*) or predicting the relationship among these species. Due to the unavailability of these important data, the lineage species concept cannot be applied sufficiently to *Ditylenchus* phylogeny. Consequently, the typological species concept may continue to provide morphological insights, until new molecular data become available. 

The major sources of soil contamination with PPNs in cultivated lands are from previous crops, weeds, or accidental introduction with infested or infected planting material from other locations [3,26,37]. Therefore, accurate species identification is crucial, because false-positive identification can result in quarantining an entire production area, while a false negative can lead to the spread of the regulated species to other cultivated regions [8]. In this context, our study provided detailed photo documentation and description of *D. anchilisposomus*, *D. clarus*, *D. tenuidens,* and *D. valveus* along with their first molecular characterization. The recovered *Ditylenchus* species are new records for Canadian nematode fauna; they are non-regulated pests and have never been reported to cause any damage to plants. However, the economically unimportant nematode species that have remained in balance with plant hosts may emerge as parasites when species habitat, agronomic practices, choice of cultivar, or rotation cycles change. Hence, it is important to identify the often-overlooked nematode infestation issues, along with the continuous application of preventive crop protection and pest management measures.

## 4. Materials and Methods

### 4.1. Nematode Isolation and Morphological Studies

To study the diversity of plant parasitic nematodes (PPNs) associated with cultivated areas of southern Alberta, we conducted a survey in selected fields of Newell, Taber, and Lethbridge Counties. The crop growing period in Alberta is approximately 4–5 months. After the harvest in late September, the fields are usually left unsown until the next growing season in the spring. The soil samples were collected in postharvest fields in October 2021 and 2022: 30 core samples were obtained from each field using a Dutch auger, and the soil was pooled to make a composite sample. Exploratory soil sampling was also conducted on the vegetation-covered edges of the fields. The soil samples were stored at 4 °C at the University of Lethbridge (southern Alberta, Canada) until processing. Nematodes were extracted from soil samples using modified Cobb’s sieving and the flotation–centrifugation method [61]. Among all soil nematodes, specimens that resembled *Ditylenchus* taxa were hand-picked individually and mounted on slides for observation and preservation. For live examination, fresh specimens of each species were transferred to a drop of distilled water, heat-relaxed, and observed under a Zeiss Axioskope 40 microscope. For morphometric studies, the nematodes were fixed, and permanent slides were prepared as described by Seinhorst [62] and De Grisse [63]. The permanent slides of studied species are stored in the Department of Biological Sciences at the University of Lethbridge. Images of each specimen were acquired using a Zeiss Axioskope 40 microscope equipped with a Zeiss Axiocam 208 camera (Carl Zeiss, Jena, Germany). Measurements from the images were performed using ZEN 3.1 (blue edition) imaging software (Carl Zeiss).

### 4.2. DNA Extraction, PCR, and Sequencing

After microscopic examination, female specimens of each species were transferred to a 0.2 mL PCR tube, and the DNA was extracted as described in Maria et al. [64]. Three sets of DNA primers (Integrated DNA Technologies, Coralville, IA, USA) were used to amplify the 18S, 28S, and ITS ribosomal RNA (rRNA) genes. The partial 18S rRNA gene sequence was amplified with the 1813F and 2646R primers [65]. The 28S rRNA gene was amplified using the D2A and D3B Primers [66], and the ITS gene was amplified using the F194 [67] and AB28R primers [68]. For the 18S, 28S, and ITS genes, the PCR conditions were as described previously [65,66,67,68]. Amplified PCR products were resolved by electrophoresis in 1% agarose gels and visualized by staining with GelRed (Biotium, Fremont, CA, USA). Amplified DNA fragments were purified using a GeneJET PCR Purification Kit (Thermo Fisher Scientific Baltics UAB, Vilnius, Lithuania) following the manufacturer’s instructions, ligated into the pJET1.2 vector (Thermo Fisher Scientific, Mississauga, ON, Canada), and introduced into competent *Escherichia coli* DH5α cells (Thermo Fisher Scientific). The presence of the PCR-derived inserts in the plasmids from transformed *E. coli* cells was confirmed with PCR. Plasmid DNA was isolated and purified using a GeneJET Plasmid Miniprep Kit (Thermo Fisher Scientific Baltics UAB) according to the manufacturer’s instructions, and sent to Azenta Inc. for DNA sequencing (South Plainfield, NJ, USA). DNA sequences were aligned using the Bioedit sequence alignment tool and compared for similarities with all known nematode species sequences in the GenBank database. 

### 4.3. Phylogenetic Studies

The DNA sequences of the 18S rRNA, 28S rRNA, and ITS rRNA genes were obtained for each *Ditylenchus* species. Newly obtained sequences and other known *Ditylenchus* species’ DNA sequences present in GenBank were used for phylogenetic analysis. The selection of outgroup taxa for each dataset was based on published studies [6,10,11,60]. Multiple nucleotide sequence alignments for the different genes were performed using the heuristics progressive method FFT-NS-2 algorithm of MAFFT v7.450 [69]. The BioEdit v7.2.5 program [70] was used for sequence alignment visualization and manually edited and trimmed of the poorly aligned positions, using a light-filtering strategy (up to 20% of alignment positions). The latter likely has little impact on tree accuracy and may save some computation time as suggested by Tan et al. [71], since methods for the automated filtering of multiple sequence alignments frequently worsen single-gene phylogenetic inference [71]. Phylogenetic analyses of the sequence datasets were performed using Bayesian inference (BI) in MrBayes v3.1.2. The best-fit model of DNA evolution was achieved using JModelTest v2.1.7 [72] with the Akaike information criterion (AIC). Accordingly, the selected models were: (1) the general time-reversible model with invariable sites and a gamma-shaped distribution (GTR + I + G) for partial 18S and D2–D3 of 28S rRNA, and the transition model with a gamma-shaped distribution (TIM2 + G) for the ITS. The best-fit model, base frequency, proportion of invariable sites, gamma distribution shape parameters, and substitution rates in the AIC were then used in MrBayes for the phylogenetic analyses, which ran with four chains for 4 × 10^6^ generations in all datasets. The sampling for Markov chains was carried out at intervals of 100 generations. For each analysis, two runs were conducted. After discarding 30% of the samples for burn-in and evaluating convergence, the remaining samples were retained for more in-depth analyses. The topologies were used to generate a 50% majority rule consensus tree. On each appropriate clade, posterior probabilities (PP) were calculated. FigTree software v.1.4.3 [73] was used for the visualization of the phylogenetic trees from all analyses.

## 5. Conclusions

There is a constant threat of the emergence of new nematode pest species. Crop rotation is considered an effective part of a management plan to mitigate some of these risks. Therefore, it is important to have good knowledge of the dominating nematode species inhabiting soil, particularly when it comes to migratory endoparasitic nematodes, such as *Ditylenchus*, which spend most of their life cycle inside plant tissues. These nematodes can be spread unknowingly and escape timely detection. In the present study, we detected four *Ditylenchus* species in no-till fields. This is the first record of any *D*. *triformis*-group species from southern Alberta, as well as their first molecular characterization. Our light micrographs and sequence-based information will enable the accurate and prompt identification of these species. In Canada, the research on the *Ditylenchus* genus is centered on *D. dipsaci* and *D. weischeri* [21,26,35]. The present work emphasizes the need for studying and documenting other *Ditylenchus* species, since many nematodes that we currently consider as benign or nonparasitic may become pests due to changes in agricultural or environmental conditions.

## Figures and Tables

**Table 1:** Comparative morphometrics of all the reported populations of *Ditylenchus anchilisposomus* (female) (Tarjan) Fortuner [28,40]. All measurements are in µm and in the form: mean ± standard deviation (range), or mean (range), or mean (Nebraska isolate).

	This Study	Tarjan [40]	Thorne and Malek [31]	Hashemi and Karegar [16]
Location	Southern Alberta, Canada	California, USA	Nebraska, USA	Southern Provinces, Iran
Habitat	No-till field, previously planted with potatoes	Grass rhizosphere	–	–
n	15	19	1	7
Body length	706.0 ± 65.3 (600.0–815.0)	624 (487–728)	640	660 (615–708)
a	40.0 ± 2.4 (36.0–43.0)	33.9 (30.6–39.9)	31	42.1 (37.3–46.9)
b	6.0 ± 0.6 (4.7–7.3)	4.7 (4.1–5.1)	6.2	5.7 (4.4–6.5)
c	13.0 ± 0.7 (12.0–14.0)	12.3 (11.1–13.2)	13	11.9 (10.6–13.2)
c’	4.5 ± 0.5 (3.6–5.5)	–	–	5.3 (4.8–5.7)
MB	37.5 ± 2.9 (31.5–42.0)	–	–	
V	82.0 ± 1.2 (81.0–85.0)	81 (78–83)	82	80.1 (77.5–82.0)
V’	92.0 ± 0.4 (91.5–93.0)	–	–	87.5 (85.3–88.7)
Lip height	2.0 ± 0.2 (2.0–3.0)	–	–	–
Lip width	6.0 ± 0.5 (5.5–7.0)	–	–	–
Stylet length	8.6 ± 0.5 (8.0–9.5)	8.8 (7.6–10.8)	10	8.4 (7.5–9)
Median bulb length	14.0 ± 1.4 (12.0–17.0)	–	–	–
Median bulb width	6.5 ± 0.5 (6.0–7.5)	–	–	–
Distance from anterior end to secretory–excretory pore	90.0 ± 4.5 (83.0–97.0)	81 (74–95)	–	–
Distance from secretory–excretory pore to hemizonid	3.0 ± 0.2 (3.0–4.0)	–	–	–
Pharynx length	118.0 ± 8.4 (102.0–128.0)	–	–	117 (107-139)
Max body diam.	18.0 ± 2.0 (15.0–21.0)	–	–	–
Vulva body width (VBW)	16.5 ± 1.6 (15.0–20.0)	–	–	–
Postvulval uterine sac (PUS) length	31.0 ± 2.4 (25.0–35.0)	32 (20–41)	–	–
PUS/VBW	1.9 ± 0.2 (1.4–2.1)	–	–	2.0 (1.4–2.6)
PUS/V–A%	45.0 ± 6.5 (33.0–54.0)	–	–	40.4 (29.2–46.2)
Distance from vulva to tail terminus	125.0 ± 9.4 (111.0–140.0)	–	–	–
Distance from vulva to anus (V–A)	69.0 ± 6.7 (60.0–82.0)	–	–	–
Anal body width (ABW)	12.0 ± 1.6 (10.0–15.0)	–	–	–
Tail length	55.5 ± 4.8 (50.0–68.0)	–	–	55.8 (51–66)

Abbreviations: n, number of specimens on which the measurements are based; a, body length/greatest body diameter; b, body length/distance from anterior end to pharyngo-intestinal junction; c, body length/tail length; c’, tail length/tail diameter at anus; MB, distance between the anterior end of the body and center of the median pharyngeal bulb as a percentage (%) of the pharynx length; V, distance from the body anterior end to the vulva as a percentage (%) of the body length; V’, the position of the vulva as a percentage (%) of the head–anus body distance.

**Table 2:** Comparative morphometrics of female *Ditylenchus clarus* Thorne and Malek [31] reported in this study and in the original description. All measurements are in µm and in the form: mean ± standard deviation (range) (southern Alberta) or mean (South Dakota).

	This Study	Thorne and Malek [31]
Location	Southern Alberta, Canada	South Dakota, USA
Habitat	*Amaranthus* sp.	–
n	20	1
Body length	540.0 ± 44.0 (452.0–610.0)	700
a	29.0 ± 2.6 (25.0–36.0)	27
b	6.0 ± 0.5 (5.0–7.0)	5.3
c	12.5 ± 0.9 (11.0–14.0)	12.8
c’	3.7 ± 0.3 (3.3–4.2)	–
MB	37.0 ± 1.3 (35.0–39.0)	–
V	82.0 ± 1.2 (79.0–85.0)	79
V’	92.0 ± 0.6 (91.0–93.0)	–
Lip height	6.2 ± 0.5 (5.0–7.0)	–
Lip width	2.5 ± 0.2 (2.0–3.0)	–
Stylet length	7.1 ± 0.1 (7.0–7.5)	10
Median bulb length	10.0 ± 1.2 (8.0–11.6)	–
Median bulb width	6.3 ± 0.6 (5.0–7.5)	–
Distance from anterior end to secretory–excretory pore	71.0 ± 2.8 (66.0–76.0)	–
Pharynx length	88.0 ± 4.8 (78.0–95.0)	–
Max body diam.	18.5 ± 1.5 (16.0–21.0)	–
Vulva body width (VBW)	17.0 ± 1.5 (15.0–19.5)	–
Postvulval uterine sac (PUS) length	17.0 ± 1.7 (15.0–21.0)	–
PUS/VBW	1.0 ± 0.1 (0.8–1.3)	–
PUS/V–A%	33.0 ± 3.5 (27.5–38.8)	–
Distance from vulva to tail terminus	95.5 ± 8.5 (79.0–112.0)	–
Distance from vulva to anus (V–A)	52.0 ± 6.2 (40.0–64.0)	–
Anal body width	12.0 ± 1.0 (10.0–13.8)	–
Tail length	43.0 ± 3.0 (39.0–48.0)	–

Abbreviations: n, number of specimens on which the measurements are based; a, body length/greatest body diameter; b, body length/distance from anterior end to pharyngo-intestinal junction; c, body length/tail length; c’, tail length/tail diameter at anus; MB, distance between the anterior end of the body and center of the median pharyngeal bulb as a percentage (%) of the pharynx length; V, distance from the body anterior end to the vulva as a percentage (%) of the body length; V’, the position of the vulva as a percentage (%) of the head–anus body distance.

**Table 3:** Morphometrics of *Ditylenchus tenuidens* Gritsenko [41] found in this study. All measurements are in µm and in the form: mean ± standard deviation (range).

Character	Female	Male
n	14	7
Body length	854.0 ± 116.1 (702.0–1091.0)	804.0 ± 55.4 (714.0–864.0)
a	42.0 ± 2.5 (38.0–47.0)	45.0 ± 6.3 (36.0–53.5)
b	6.0 ± 0.7 (5.0–7.6)	6.0 ± 0.5 (5.0–6.5)
c	9.0 ± 0.9 (8.0–11.0)	9.0 ± 0.9 (8.0–11.0)
c’	7.0 ± 0.8 (5.4–8.5)	6.0 ± 0.6 (6.0–7.0)
MB	40.0 ± 2.4 (36.0–46.0)	40.0 ± 1.8 (37.0–41.0)
V	79.0 ± 2.1 (74.0–82.5)	–
V’	89.0 ± 1.0 (80.3–90.0)	–
Lip height	3.0 ± 0.8 (2.0–5.5)	6.0 ± 0.4 (5.0–6.5)
Lip width	6.0 ± 0.5 (5.5–7.5)	2.3 ± 0.3 (2.0–3.0)
Stylet length	8.4 ± 0.5 (8.0–9.5)	8.4 ± 0.3 (8.0–9.0)
Median bulb length	14.0 ± 1.5 (11.0–17.0)	12.6 ± 1.6 (11.0–15.0)
Median bulb width	9.0 ± 1.0 (8.0–10.5)	8.0 ± 1.3 (7.0–10.0)
Distance from anterior end to secretory–excretory pore	105.0 ± 7.1 (90.0–112.0)	103.0 ± 2.1 (101.0–106.0)
Distance from secretory–excretory pore to hemizonid	3.6 ± 0.3 (3.0–4.0)	3.7 ± 0.3 (3.2–4.0)
Pharynx length	139.0 ± 7.0 (126.0–148.0)	138.0 ± 5.0 (132.0–146.0)
Max body diam.	20.0 ± 2.4 (17.0–24.0)	18.0 ± 2.6 (15.0–21.0)
Vulva body width (VBW)	18.0 ± 1.3 (16.0–21.0)	–
Postvulval uterine sac (PUS) length	22.0 ± 3.2 (18.0–28.0)	–
PUS/VBW	1.2 ± 0.2 (1.0–1.5)	–
PUS/V–A%	28.0 ± 3.7 (17.5–32.0)	–
Distance from vulva to tail terminus	175.0 ± 24.3 (137.0–212.0)	–
Distance from vulva to anus (V–A)	82.0 ± 17.0 (60.0–119.0)	–
Anal/cloacal body width	13.5 ± 1.4 (11.0–16.0)	14.0 ± 1.1 (13.0–16.0)
Tail length	94.0 ± 9.9 (75.0–107.0)	89.0 ± 7.2 (75.0–96.0)
Spicule length	–	17.0 ± 1.9 (15.0–20.0)
Gubernaculum length	–	6.0 ± 0.7 (5.0–6.5)

Abbreviations: n, number of specimens on which the measurements are based; a, body length/greatest body diameter; b, body length/distance from anterior end to pharyngo-intestinal junction; c, body length/tail length; c’, tail length/tail diameter at anus; MB, distance between the anterior end of the body and center of the median pharyngeal bulb as a percentage (%) of the pharynx length; V, distance from the body anterior end to the vulva as a percentage (%) of the body length; V’, the position of the vulva as a percentage (%) of the head–anus body distance.

**Table 4:** Comparative morphometrics of all the reported populations of *Ditylenchus tenuidens* (female) Gritsenko [41]. All measurements are in µm and in the form: mean (range), or range.

	This Study	Gritsenko [41]	Brzeski [27]				Mirshekari and Abdollahi [45]
Location	Southern Alberta, Canada	Kyrgyzstan	Bialowieska National Park, Poland			Sweden	Northern Province, Iran
Habitat	No-till field previously planted with grains	Potato, winter wheat	–	Coniferous litter	Moss	Moss	Vegetables, wheat
n	14	–	8	10 ^a^	4	11 ^a^	5
Body length	854.0 (702–1091)	–	646 (591–677)	550–853	665 (625–732)	552–770	644 (531–738)
a	42.0 (38–47)	–	35 (29–40)	31–44	37 (32–42)	34–42	42 (38.6–46.2)
b	6.0 (5.0–7.6)	–	4.6–5.0	4.1–6.8	4.9 (4.6–5.1)	4.4–4.9	6.1 (5.2–7.2)
c	9.0 (8.0–11.0)	9.0–12.9	10.3 (9.5–12.7)	10.1–11.6	10.7 (10–11.2)	9.7–12.2	12 (10.8–13.9)
c’	7.0 (5.4–8.5)	4.1–6.8	5.5 (4.4–6.0)	4.6–6.0	5.8 (5.2–6.8)	4.7–6.2	5.1 (4.4–6.0)
MB	40.0 (36–46)	–	36–41	38–42	39–41	36–39	45.8 (37.5–55.2)
V	79.0 (74–82.5)	76–82	77–79	75–82	77–79	76–79	79.9 (74.3–84)
V’	89.0 (80.3–90)	–	85–88	77–87	85-86	84–87	–
Stylet length	8.4 (8.0–9.5)	7–9	8	7.0–8.5	8	7.5–8.5	7.8 (7.5–8.1)
Distance from anterior end to secretory–excretory pore	105.0 (90–112)	–	93 (87–97)	81–103	91–101	85–93	88.3 (79–98)
Pharynx length	139.0 (126–148)	–	134 (128–141)	111–135	136 (129–145)	126–146	106 (90–120)
Postvulval uterine sac (PUS) length	22.0 (18–28)	18–37					25.1 (15–32.5)
Tail length	94.0 (75–107)		64 (53–69)	47–95	63 (56–73)	62	43.8 (49.3–60)

^a^ Composite values of two populations. Abbreviations: n, number of specimens on which the measurements are based; a, body length/greatest body diameter; b, body length/distance from anterior end to pharyngo-intestinal junction; c, body length/tail length; c’, tail length/tail diameter at anus; MB, distance between the anterior end of the body and center of the median pharyngeal bulb as a percentage (%) of the pharynx length; V, distance from the body anterior end to the vulva as a percentage (%) of the body length; V’, the position of the vulva as a percentage (%) of the head–anus body distance.

**Table 5:** Morphometric characteristics of *Ditylenchus valveus* Thorne and Malek [31] found in this study. All measurements are in µm and in the form: mean ± standard deviation (range).

Character	Females	Males
n	45	5
Body length	976.0 ± 64.4 (863.0–1097.0)	810.0 ± 7.1 (800.0–819.0)
a	40.5 ± 4.3 (34.0–54.5)	44.0 ± 4.7 (38.5–50.0)
b	7.0 ± 0.5 (5.7–8.1)	6.2 ± 0.1 (6.1–6.4)
c	10.5 ± 0.8 (8.0–12.0)	9.5 ± 1.1 (9.0–11.5)
c’	6.0 ± 0.5 (5.2–7.7)	6.5 ± 1.2 (5.0–8.0)
MB	37.0 ± 2.3 (29.5–42.0)	38.7 ± 0.4 (38.0–39.0)
V/T	82.0 ± 1.2 (79.0–84.0)	62.0 ± 3.9 (59.0–65.0)
V’	91.0 ± 0.8 (88.0–92.0)	–
Lip height	2.4 ± 0.2 (2.0–3.0)	2.1 ± 0.1 (2.0–2.5)
Lip width	5.9 ± 0.4 (5.0–7.0)	5.4 ± 0.2 (5.0–6.0)
Stylet length	8.2 ± 0.3 (7.0–9.0)	7.7 ± 0.4 (7.0–8.0)
Median bulb length	13.5 ± 1.4 (11.0–16.0)	12.0 ± 2.0 (10.0–14.0)
Median bulb width	7.5 ± 0.7 (6.0–9.0)	6.7 ± 0.6 (6.0–8.0)
Distance from anterior end to secretory–excretory pore	114.0 ± 6.3 (101.131.0)	103.0 ± 2.1 (100.0–105.0)
Distance from secretory–excretory pore to hemizonid	3.8 ± 0.6 (2.6–5.0)	3.5 ± 0.7 (3.0–4.0)
Pharynx length	143 ± 9.7 (130.0–170.0)	130.4 ± 1.8 (128.0–133.0)
Max body diam.	24.5 ± 2.4 (20.0–30.0)	18.4 ± 2.1 (16.0–21.0)
Vulva body width (VBW)	22.0 ± 1.5 (19.0–25.0	–
Postvulval uterine sac (PUS) length	20.5 ± 2.7 (15.0–26.0)	–
PUS/VBW	0.9 ± 0.1 (0.6–1.2)	–
PUS/V–A%	25.5 ± 3.6 (19.0–37.0)	–
Distance from vulva to tail terminus	175.0 ± 10.3 (143.0–194.0)	–
Distance from vulva to anus (V–A)	81.0 ± 6.3 (63.0–94.0)	–
Anal body width (ABW)	15.7 ± 1.1 (13.5–18.0)	–
Cloacal body width	–	13.6 ± 2.7 (11.0–18.0)
Tail length	93.0 ± 5.4 (80.0–108.0)	85.8 ± 8.5 (71.0–92.0)
Spicule length	–	20.3 ± 1.0 (19.0–21.0)
Gubernaculum length	–	6.2 ± 0.3 (6.0–6.5)

Abbreviations: n, number of specimens on which the measurements are based; a, body length/greatest body diameter; b, body length/distance from anterior end to pharyngo-intestinal junction; c, body length/tail length; c’, tail length/tail diameter at anus; MB, distance between the anterior end of the body and center of the median pharyngeal bulb as a percentage (%) of the pharynx length; V, distance from the body anterior end to the vulva as a percentage (%) of the body length; V’, the position of the vulva as a percentage (%) of the head–anus body distance; T, distance from cloacal aperture to anterior end of testis as a percentage (%) of the body length.

**Table 6:** Comparative morphometrics of all the reported populations of *Ditylenchus valveus* (female) Thorne and Malek [31]. All measurements are in µm and in the form: the mean (range) or mean (South Dakota).

	This Study	Thorne and Malek [31]	Anderson [29]	Brzeski [27]		Karani et al. [48]	Hashemi and Karegar [16]
Location	Southern Alberta, Canada	South Dakota, USA	Manitoba, Canada	Oltarzew, Poland	Varna, Bulgaria	Northern Province, Iran	Southern Provinces, Iran
Habitat	No-till field, previously planted with barley	Plowed field	Mushroom compost	Cultivated loamy soil	Rhizosphere of unidentified bush	Olive tree rhizosphere	–
n	45	1	6	7	5	9	9
Body length	976.0 (863.0–1097.0)	900	883 (736–1088)	910 (831–998)	677 (621–725)	970 (919–1037)	739 (638–907)
a	40.5 (34.0–54.5)	36	42 (37–45)	42 (35–52)	41 (35–46)	32.5 (25–36.5)	38.3 (29.3–51.7)
b	7.0 (5.7–8.1)	7.4	7.3 (5.3–8.9)	6.8 (6.0–7.8)	5.5 (5.0–5.8)	6.2 (5.7–6.9)	6.1 (5.2–8.0)
c	10.5 (8.0–12.0)	12	9.6 (8.4–10.9)	9.9 (9.6–10.3)	11.4 (10.2–14.1)	11.1 (9.0–12.0)	11.5 (9.8–14.4)
c’	6.0 (5.2–7.7)	–	7.5 (5.8–8.8)	7.0 (6.8–7.2)	5.7 (5.0–6.3)	5.2 (4.5–6.4)	5.5 (4.5–7.4)
MB	37.0 (29.5–42.0)	–	–	37 (35–38)	–	36.0 (34.0–37.7)	80.4 (79.0–82.7)
V	82.0 (79.0–84.0)	81	81 (80–82)	80–82	79–82	79.1 (76–81)	–
Stylet length	8.2 (7.0–9.0)	12	8 (7–9)	7.0–7.5	7.5 (7–8)	7.7 (7–8)	7.2 (7–8)
Pharynx length	143 (130.0–170.0)	–	116–139	135 (128–144)	122 (118–131)	157 (146–164)	123 (106–153)
Distance from anterior end to secretory–excretory pore	114.0 (101.0-131.0)	–	97–108	106 (101–111)	90 (83–100)	116 (107–125)	–
Max body diam.	24.5 (20.0–30.0)	–	16–22	–	–	–	–
Postvulval uterine sac (PUS) length	20.5 (15.0–26.0)	–	23–36	–	–	46 (40–52)	–
Distance from vulva to anus	81.0 (63.0–94.0)	–	79–91	–	–	–	–
Tail length	93.0 (80.0–108.0)	–	59–88	92 (80–99)	60 (52–69)	88 (77–102)	66.4 (55–92)
Spicule length	–	–	19– 20	20	18.5 (16–23)	22.8 (22–23.5)	18.5–19
Gubernaculum length	–	–	5–6	–	–	8	–

Abbreviations: n, number of specimens on which the measurements are based; a, body length/greatest body diameter; b, body length/distance from anterior end to pharyngo-intestinal junction; c, body length/tail length; c’, tail length/tail diameter at anus; MB, distance between the anterior end of the body and center of the median pharyngeal bulb as a percentage (%) of the pharynx length; and V, distance from the body anterior end to the vulva as a percentage (%) of the body length.

## Data Availability

Not applicable.

## References

[1] Filipjev I. (1936). On the classification of the Tylenchinae. Proc. Helminthol. Soc. Wash..

[2] Nicoll W. (1935). VI. Vermes. Rhabditida. Anguinidae. Zool. Rec..

[3] Siddiqi M.R. (2000). Tylenchida: Parasites of Plants and Insects.

[4] Subbotin S.A., Madani M., Krall E., Sturhan D., Moens M. (2005). Molecular diagnostics, taxonomy, and phylogeny of the stem nematode *Ditylenchus dipsaci* species complex based on the sequences of the internal transcribed spacer-rDNA. Phytopathology.

[5] Nicol J., Turner S., Coyne D.L., Nijs L.d., Hockland S., Maafi Z.T. (2011). Current nematode threats to world agriculture. Genomics and Molecular Genetics of Plant-Nematode Interactions.

[6] Vovlas N., Troccoli A., Palomares-Rius J.E., De Luca F., Liébanas G., Landa B.B., Subbotin S.A., Castillo P. (2011). *Ditylenchus gigas* n. sp. parasitizing broad bean: A new stem nematode singled out from the *Ditylenchus dipsaci* species complex using a polyphasic approach with molecular phylogeny. Plant Pathol..

[7] Goodey J. (1958). *Ditylenchus myceliophagus* n. sp.(Nematoda: Tylenchidae). Nematologica.

[8] Perry R.N., Moens M. (2013). Plant Nematology.

[9] Zhang S., Liu G., Janssen T., Zhang S., Xiao S., Li S., Couvreur M., Bert W. (2014). A new stem nematode associated with peanut pod rot in China: Morphological and molecular characterization of *Ditylenchus arachis* n. sp.(Nematoda: Anguinidae). Plant Pathol..

[10] Giblin-Davis R.M., Erteld C., Kanzaki N., Ye W., Zeng Y., Center B.J. (2010). *Ditylenchus halictus* n. sp.(Nematoda: Anguinidae), an associate of the sweat bee, *Halictus sexcinctus* (Halictidae), from Germany. Nematology.

[11] Xue Q., Slonim O., Bucki P., Mendel Z., Protasov A., Golan O., Vieira P., Miyara S.B. (2019). Diversity and distribution of nematodes associated with bark beetles in Israel. Nematology.

[12] Oliveira R.D., Santin Â.M., Seni D.J., Dietrich A., Salazar L.A., Subbotin S.A., Mundo-Ocampo M., Goldenberg R., Barreto R.W. (2013). *Ditylenchus gallaeformans* sp. n.(Tylenchida: Anguinidae)—A neotropical nematode with biocontrol potential against weedy Melastomataceae. Nematology.

[13] Hawn E. (1963). Transmission of bacterial wilt of alfalfa by *Ditylenchus dipsaci* (Kühn). Nematologica.

[14] Hawn E. (1973). Plant-parasitic nematodes in irrigated soils of Alberta. Can. Plant Dis. Surv..

[15] Moens M., Perry R.N. (2009). Migratory plant endoparasitic nematodes: A group rich in contrasts and divergence. Annu. Rev. Phytopathol..

[16] Hashemi K., Karegar A. (2019). Description of *Ditylenchus paraparvus* n. sp. from Iran with an updated list of *Ditylenchus* Filipjev, 1936 (Nematoda: Anguinidae). Zootaxa.

[17] Wendt K., Swart A., Vrain T., Webster J. (1995). *Ditylenchus africanus* sp. n. from South Africa; a morphological and molecular characterization. Fundam. Appl. Nematol..

[18] Butler E.J. (1913). Diseases of Rice.

[19] Thorne G. (1945). *Ditylenchus destructor* n. sp. the potato rot nematode, and *Ditylenchus dipsaci* (Kühn, 1857) Filipjev, 1936, the teasel nematode (Nematoda: Tylenchidae). Proc. Helminthol. Soc. Wash..

[20] Kühn J. (1857). Über das Vorkommen von Anguillulen in erkrankten Blüthenköpfen von Dipsacus fullonum L. Z. Für Wiss. Zool..

[21] Madani M., Tenuta M., Chizhov V.N., Subbotin S.A. (2015). Diagnostics of stem and bulb nematodes, *Ditylenchus weischeri* and *D. dipsaci* (Nematoda: Anguinidae), using PCR with species-specific primers. Can. J. Plant Pathol..

[22] Yaghoubi A., Pourjam E., Ye W., Castillo P., Pedram M. (2018). Description and molecular phylogeny of *Ditylenchus gilanicus* n. sp.(Nematoda: Anguinidae) from northern forests of Iran. Eur. J. Plant Pathol..

[23] Arriola Í.A., dos Santos Isaias R.M. (2021). Extending the knowledge on the histological patterns of leaf galls induced by *Ditylenchus gallaeformans* (Nematoda) on Miconia (Melastomataceae) hosts. Flora.

[24] Castillo P. (2022). Ditylenchus Gigas (Giant Stem and Bulb Nematode). CABI Compend Datasets.

[25] Prasad J., Varaprasad K. (2002). Ufra nematode, *Ditylenchus angustus* is seed borne!. Crop Prot..

[26] Hajihassani A., Tenuta M., Gulden R.H. (2016). Host preference and seedborne transmission of *Ditylenchus weischeri* and *D. dipsaci* on select pulse and non-pulse crops grown in the Canadian Prairies. Plant Dis..

[27] Brzeski M.W. (1991). Review of the genus *Ditylenchus* filipjev, 1936 (Nematoda: Anguinidae). Rev. Nématologie.

[28] Fortuner R. (1982). On the genus *Dilylenchus* Filipjev, 1936 (Nernatoda: Tylenchida). Rev. Nematol..

[29] Anderson R. (1983). An emended description of *Ditylenchus valveus* Thorne &Malek, 1968 and description of *D. filimus* n. sp.(Nematoda: Tylenchidae) from mushroom compost in Canada. Can. J. Zool..

[30] Anderson R., Mulvey R. (1980). Description, relationships, and host symptoms of *Ditylenchus dryadis* n. sp.(Nematoda: Tylenchidae) from the Canadian High Arctic, a transitional species of gall-forming parasite attacking *Dryas integrifolia* M. Vahl. Can. J. Zool..

[31] Thorne G., Malek R.B. (1968). Nematodes of the Northern Great Plains: Part 1 Tylenchida [Nemata Secernentra]. South Dak. Agric. Exp. Stn. Tech. Bull..

[32] Chizhov V.N., Borisov B.A., Subbotin S.A. (2010). A new stem nematode, *Ditylenchus weischeri* sp. n.(Nematoda: Tylenchida), a parasite of *Cirsium arvense* (L.) Scop. in the central region of the non-chernozem zone of Russia. Russ. J. Nematol..

[33] Yu Q., Ye W., Badiss A., Sun F. (2010). Description of *Ditylenchus dipsaci* (Kuhn, 1857) Filipjev, 1936 (Nematoda: Anguinidae) infesting garlic in Ontario, Canada. Int. J. Nematol..

[34] Yu Q., Zaida M., Hughes B., Celetti M. (2012). Discovery of potato rot nematode, *Ditylenchus destructor*, infesting garlic in Ontario, Canada. Plant Dis..

[35] Tenuta M., Madani M., Briar S., Molina O., Gulden R., Subbotin S.A. (2014). Occurrence of *Ditylenchus weischeri* and not *D. dipsaci* in field pea harvest samples and *Cirsium arvense* in the Canadian Prairies. J. Nematol..

[36] Poirier S., Dauphinais N., Van Der Heyden H., Véronneau P.-Y., Bélair G., Gravel V., Mimee B. (2019). Host range and genetic characterization of *Ditylenchus dipsaci* populations from eastern Canada. Plant Dis..

[37] Munawar M., Yevtushenko D.P., Palomares-Rius J.E., Castillo P. (2021). Species diversity of pin nematodes (*Paratylenchus* spp.) from potato growing regions of southern Alberta, Canada. Plants.

[38] Munawar M., Yevtushenko D.P., Castillo P. (2021). Integrative taxonomy, distribution, and host associations of *Geocenamus brevidens* and *Quinisulcius capitatus* and from southern Alberta, Canada. J. Nematol..

[39] Munawar M., Castillo P., Yevtushenko D.P. (2022). Description of *Filenchus* species from agroecosystem of southern Alberta, Canada. Agronomy.

[40] Tarjan A. (1958). A new genus, *Pseudhalenchus* (Tylenchinae: Nematoda), with descriptions of two new species. Proc. Helminthol. Soc. Wash..

[41] Gritsenko V. (1971). *Ditylenchus tenuidens* n. sp. and *Aphelenchoides curiolis* n. sp.(Nematoda, Tylenchidae and Aphelenchoididae) from Kirgizia. Zool. Zhurnal.

[42] Cobb M.V. (1933). New nemic genera and species, with taxonomic notes. J. Parasitol..

[43] Bastian H.C. (1865). Monograph on the Anguillulidae, or Free Nematoids, Marine, Land, and Freshwater; with Descriptions of 100 New Species. Trans. Linn. Soc. Lond..

[44] Thorne G. (1961). Principles of Nematology.

[45] Mirshekari K., Abdollahi M. (2015). Description of two known species of *Ditylenchus,* collected from wheat fields of Boyer-Ahmad and Dena regions, Kohgiluyeh and Boyer-Ahmad Province. Iran. J. Plant Pathol..

[46] Brzeski M.W. (1967). The effect of host on morphology and population increase of *Ditylenchus myceliophagus* Goodey (Nematoda: Tylenchidae). Bull. L’academie Pol. Sciences. Cl. II. Ser. Sci. Biol..

[47] Evans A., Fisher J. (1970). The effect of environment on nematode morphometrics. Comparison of *Ditylenchus myceliophagus* and *D. destructor*. Nematologica.

[48] Karani H.M., Eskandari A., Ghaderi R., Heydari R., Miraeez E. (2017). Morphological characterisation of a new and two known species of *Ditylenchus* Filipjev, 1936 (Nematoda: Anguinidae) from Iran. Zootaxa.

[49] Steiner G., Buhrer E.M. (1934). *Aphelenchoides xylophilus* n. sp., a nematode associated with blue-stain and other fungi in timber. J. Agric. Res..

[50] Nickle W.R. (1970). A taxonomic review of the genera of the Aphelenchoidea (Fuchs, 1937) Thorne, 1949 (Nematoda: Tylenchida). J. Nematol..

[51] Ritzema Bos J. (1890). De bloemkoolziekte der aardbeien, veroorzaakt door Aphelenchus fragariae nov. spec. Voorloopige Meded. Maanblad Nat..

[52] Christie J. (1932). Recent observations on the strawberry dwarf neaiatode in Massachusetts. Plant Dis. Rep..

[53] Siddiqi M.R. (1963). Four new species in the sub-family Tylenchinae (Nematoda) from North India. Z. Fur Parasitenkd..

[54] Zhao L., Ye W., Maria M., Pedram M., Gu J. (2017). *Xiphinema japonicum* n. sp.(Nematoda: Longidorinae) from the Rhizosphere of Japanese *Podocarpus macrophyllus* (Thunb.), a cryptic species related to *Xiphinema bakeri* Williams, 1961. J. Nematol..

[55] Lehman P.S. (1994). Dissemination of phytoparasitic nematodes. Nematol. Circ..

[56] Forge T.A., Larney F.J., Kawchuk L.M., Pearson D.C., Koch C., Blackshaw R.E. (2015). Crop rotation effects on *Pratylenchus neglectus* populations in the root zone of irrigated potatoes in southern Alberta. Can. J. Plant Pathol..

[57] Orley L. (1880). Az anguillulidák magánrajza (Monographie der Anguilluliden). Természetrajzi Füzetek Bp..

[58] Jones J.T., Haegeman A., Danchin E.G., Gaur H.S., Helder J., Jones M.G., Kikuchi T., Manzanilla-López R., Palomares-Rius J.E., Wesemael W.M. (2013). Top 10 plant-parasitic nematodes in molecular plant pathology. Mol. Plant Pathol..

[59] Somasekhar N., Prasad J.S. (2012). Plant–nematode interactions: Consequences of climate change. Crop Stress and Its Management: Perspectives and Strategies.

[60] Skwiercz A.T., Kornobis F.W., Winiszewska G., Przybylska A., Obrępalska-Stęplowska A., Gawlak M., Subbotin S.A. (2017). *Ditylenchus laurae* sp. n.(Tylenchida: Anguinidae) from Poland–a new species of the *D. dipsaci* complex associated with a water plant, *Potamogeton perfoliatus* L.. Nematology.

[61] Jenkins W. (1964). A rapid centrifugal-flotation technique for separating nematodes from soil. Plant Dis. Report..

[62] Seinhorst J. (1959). A rapid method for the transfer of nematodes from fixative to anhydrous glycerin. Nematologica.

[63] De Grisse A.T. (1969). Redescription ou Modifications de Quelques Technique Utilis dan L’etude des Nematodes Phytoparasitaires.

[64] Maria M., Powers T., Tian Z., Zheng J. (2018). Distribution and description of criconematids from Hangzhou, Zhejiang Province, China. J. Nematol..

[65] Holterman M., van der Wurff A., van den Elsen S., van Megen H., Bongers T., Holovachov O., Bakker J., Helder J. (2006). Phylum-wide analysis of SSU rDNA reveals deep phylogenetic relationships among nematodes and accelerated evolution toward crown clades. Mol. Biol. Evol..

[66] De Ley P., Felix M.-A., Frisse L., Nadler S., Sternberg P., Thomas W.K. (1999). Molecular and morphological characterisation of two reproductively isolated species with mirror-image anatomy (Nematoda: Cephalobidae). Nematology.

[67] Ferris V., Ferris J., Faghihi J. (1993). Variation in spacer ribosomal DNA in some cyst-forming species of plant parasitic nematodes. Fundam. Appl. Nematol..

[68] Curran J., Driver F., Ballard J., Milner R. (1994). Phylogeny of Metarhizium: Analysis of ribosomal DNA sequence data. Mycol. Res..

[69] Katoh K., Rozewicki J., Yamada K.D. (2019). MAFFT online service: Multiple sequence alignment, interactive sequence choice and visualization. Brief. Bioinform..

[70] Hall T.A. (1999). BioEdit: A user-friendly biological sequence alignment editor and analysis program for Windows 95/98/NT. Nucleic Acids Symposium Series.

[71] Tan G., Muffato M., Ledergerber C., Herrero J., Goldman N., Gil M., Dessimoz C. (2015). Current methods for automated filtering of multiple sequence alignments frequently worsen single-gene phylogenetic inference. Syst. Biol..

[72] Darriba D., Taboada G.L., Doallo R., Posada D. (2012). jModelTest 2: More models, new heuristics and parallel computing. Nat. Methods.

[73] Rambaut A. (2014). FigTree v1. 4.2, A Graphical Viewer of Phylogenetic Trees. http://tree.bio.ed.ac.uk/software/figtree/rightanglebracket.

